# Amino acid profiles and clinical phenotypes in young children with autism spectrum disorder

**DOI:** 10.3389/fnins.2026.1913701

**Published:** 2026-09-03

**Authors:** Ilaria Serati, Luca Lalli, Andrea Riccardo Dallapiccola, Pietro Baso, Martina Tosi, Francesca Brustia, Maria Paola Canevini, Michele Mussap, Livia Pisciotta, Emilia Ricci, Maura Rossi, Marco Di Dario, Maria Cristina Straface, Amanda Papa, Sabrina Fede, Salvatore Fazzone, Elena Spada, Federica Gioia, Roberto Longo, Pierangelo Veggiotti, Gianvincenzo Zuccotti, Cristina Cereda, Bruno Mario Cesana, Simona Ferraro

**Affiliations:** 1 Child and Adolescent Neuropsychiatry Unit, ASST Fatebenefratelli Sacco, Milan, Italy; 2 Unit of Translational Immunology, IRCCS Foundation National Cancer Institute, Milan, Italy; 3 Child Neuropsychiatry Unit, Epilepsy Center, San Paolo Hospital, Milan, Italy; 4 Department of Child Neurology and Psychiatry, IRCCS Mondino Foundation, Pavia, Italy; 5 Department of Health Sciences, University of Milan, Milan, Italy; 6 Department of Pediatrics, Buzzi Children’s Hospital, Milan, Italy; 7 Child Neuropsychiatry Unit, University Hospital Maggiore della Carità, Novara, Italy; 8 Laboratory Medicine, Hospital Foundation Villa Salus, Venice, Italy; 9 Molecular Unit, Department of Surgical Sciences, University of Cagliari, Cagliari, Italy; 10 Laboratory of Clinical Pathology, Department of Diagnostic Services, ASST Santi Paolo e Carlo, Milan, Italy; 11 Center of Functional Genomics and Rare Diseases, Buzzi Children’s Hospital, Milan, Italy; 12 Laboratorio Della Conoscenza Carlo Corchia, Florence, Italy; 13 Corporate Information Systems, Buzzi Children’s Hospital, Milan, Italy; 14 Department of Biomedical and Clinical Sciences, University of Milan, Milan, Italy; 15 Pediatric Neurology Unit, V. Buzzi Children’s Hospital, Milan, Italy; 16 Laboratory of Medical Statistics, Biometry and Epidemiology “G.A. Maccacaro”, Department of Clinical Sciences and Community Health, University of Milan, Milan, Italy

**Keywords:** amino acid, autism spectral disorder, children, clinical phenotypes, nutrition

## Abstract

**Introduction:**

In 394 children with autism spectrum disorder (ASD), we investigated the association between plasma amino acid (AA) concentrations and clinical phenotypes.

**Methods:**

Multivariable logistic regression models were used to evaluate the associations between 22 AAs and clinical features, reporting odds ratios.

**Results:**

The median age at diagnosis was 3.5 years (interquartile range: 2.8–4.6 years) for boys and 3.1 years (interquartile range: 2.3–3.8 years) for girls; 81.5% of the participants were boys. A 25-unit increase in cystine concentration was associated with stereotypies (OR = 2.07), hyperactivity (OR = 1.79), and aggressive behavior (OR = 2.13), suggesting a potential role of oxidative stress in these phenotypes. Similarly, a 25-unit increase in taurine levels was associated with hyperactivity (OR = 1.24) and intellectual disability (OR = 1.38), consistent with oxidative imbalance. Higher arginine levels appeared to have a protective effect against hyperactivity (OR = 0.70), indicating a potential role in ammonia detoxification. Increased glutamine and tyrosine levels were associated with ORs < 1 for stereotypies, supporting potential neuroprotective roles.

**Discussion:**

These findings suggest oxidative stress, urea cycle dysfunction, and altered neurotransmission may contribute to the pathophysiology of ASD and related clinical phenotypes in young children, warranting further investigation to inform the development of personalized clinical and nutritional interventions.

## 1 Introduction

Autism spectrum disorder (ASD) is a neurodevelopmental condition that affected approximately 1 in 36 children aged 8 years in 2020, with a male-to-female ratio of approximately 3.8:1 (Maenner et al., 2023). ASD is characterized by persistent deficits in social communication and social interaction, along with restricted and repetitive patterns of behavior, interests, or activities. The clinical presentation is highly heterogeneous, ranging from mild to severe impairment. Early behavioral signs that suggest ASD can be identified as early as 9 months of age (Hirota and King, 2023). ASD is considered a complex, multifactorial disorder resulting from the interplay of genetic, environmental, maternal, gestational, and other risk factors. Despite substantial advances in understanding its etiology, the majority of cases remain idiopathic (Sanders et al., 2015; Cross-Disorder Group of the Psychiatric Genomics Consortium, 2019).

Although rare, several inherited metabolic disorders may exhibit features consistent with an ASD phenotype (Žigman et al., 2021). Consequently, the exclusion of underlying metabolic conditions through appropriate laboratory investigations, such as fasting plasma amino acid analysis, urine organic acid analysis, and acylcarnitine profiling, may be warranted, particularly because some of these disorders have a defined inheritance pattern and potentially effective treatments (Ashe et al., 2019). Nevertheless, studies have shown that the diagnostic yield and cost-effectiveness of routine metabolic screening are low when applied to unselected children with ASD (Hyman et al., 2020; Campistol et al., 2016; Schiff et al., 2011). Therefore, the decision to pursue a metabolic evaluation should be guided by clinical findings and family history.

In children with ASD, the characterization of AA patterns, particularly defects in homeostasis and metabolism, is a divisive topic (Schiff et al., 2011; Ferraro et al., 2025). Current literature reports often controversial data, with some studies demonstrating increased, decreased, or unchanged concentrations of several AAs, including taurine, ornithine, arginine, glycine, and branched-chain amino acids (BCAAs), in children with ASD compared to neurotypical peers. These discrepancies likely reflect age-related metabolic changes, heterogeneity among study populations, and, in some cases, the limitations associated with univariate statistical approaches (Ferraro et al., 2025).

Relatively few studies have investigated the relationship between AA profiles and specific ASD phenotypes or symptom severity using robust multivariate statistical methods.

Accordingly, several AA abnormalities have been associated with distinct clinical features. Increased levels of tryptophan, homocysteine, and phenylalanine, along with decreased levels of methionine and tyrosine, have been linked to hyperactivity (Yektaş et al., 2019; Arum et al., 2017). Elevated glutamate concentrations and an increased glutamate-to-glutamine ratio have been proposed as metabolic signatures of ASD and have also been associated with greater disease severity (Zheng et al., 2016). Reduced BCAA levels have been linked to impairments in motor function and social behavior (Li et al., 2025). Furthermore, increased concentrations of aspartic acid, glutamic acid, phenylalanine, leucine, and isoleucine, along with decreased levels of tryptophan and valine, have been associated with more severe manifestations of ASD according to preliminary predictive models (Li et al., 2025). Notably, all of these AAs play key roles in modulating neurotransmission, maintaining the excitatory–inhibitory balance, and regulating cerebral blood flow (Maynard and Manzini, 2017). Overall, the available evidence supports the hypothesis that disturbances in AA metabolism may contribute to the development and clinical manifestations of ASD (Ferraro et al., 2025; Maynard and Manzini, 2017; Dawkins et al., 2016; James et al., 2004; Chang et al., 2024; Smith et al., 2019; Ahmed, 2025; Wang et al., 2022).

The primary aim of the present study was to characterize the association between AA profiles and major clinical phenotypes of ASD, including self-directed and outwardly directed aggressive behavior, dyspraxia, language impairment, hyperactivity, stereotyped behaviors, developmental regression, and intellectual disability, in a large cohort of very young children at the time of diagnosis. The secondary aim was to consider the potential clinical utility of AA profiling as a tool to support the identification of targeted nutritional interventions that may help mitigate ASD-related symptoms (Maynard and Manzini, 2017; Smith et al., 2019). This retrospective study was designed to strengthen the evidence provided by previous studies, which largely relied on univariate statistical analyses. By including a large cohort of children diagnosed early in life and collecting a comprehensive set of detailed clinical variables, we were able to apply multivariable statistical analyses to identify significant independent associations between AA patterns and clinical phenotypes. This approach provides a more reliable and comprehensive evaluation of these relationships specifically in young children, a population that has been underrepresented in previous studies, which generally included broader pediatric age ranges.

## 2 Materials and methods

### 2.1 Study design

This is a multicenter, observational, retrospective study with consecutive enrollment between 29 January 2016 and 30 September 2024 of a cohort of 442 children with a confirmed diagnosis of ASD, established according to Diagnostic and Statistical Manual of Mental Disorders (2026). These children underwent AA testing on plasma samples collected from fasting blood drawn at 7:00 a.m., following an overnight stay and a 1-day acclimatization period in the pediatric neurology ward, as part of their diagnostic workup for ASD. Metabolic testing is routinely performed on all children with ASD at the time of diagnosis, in accordance with the regional operational plan established by the Lombardy Regional Executive Committee (2026). The study was conducted across three neurological centers: (1) Fatebenefratelli-Buzzi Local Public Health and Social Services Unit, Milan; (2) Santi Paolo and Carlo Hospitals, Milan; and (3) Ospedale Maggiore della Carità, Novara. It involved two laboratories for AA detection (the Laboratory of Clinical Pathology at Santi Paolo and Carlo Hospitals and the Center of Functional Genomics and Rare Diseases at Buzzi Children’s Hospital in Milan).

### 2.2 Laboratory data

All procedures have been detailed in a previous study (Ferraro et al., 2025). Briefly, venous blood samples were collected into Li-heparin-coated tubes from the cubital vein in the morning, following an overnight fast, at the neurological clinical center where children diagnosed with ASD were admitted. Within 30 min of collection, the samples were centrifuged at 3500 rpm for 15 min to obtain plasma for subsequent analysis. The plasma was then sent to the laboratory and stored at 4–8 °C or frozen at −20 °C. Hemolyzed specimens were discarded. Plasma was deproteinized before analysis by mixing it with a 10% sulfosalicylic acid solution containing the required concentration of the internal standard [2-amino-2-deoxy-d-gluconic acid (SIGMA Aldrich, Merck Spa, Milan, Italy)] (Smon et al., 2019). The sample was mixed and incubated for 20 min at 4 °C to allow for the formation of a precipitate. After centrifuging at 3500 rpm for 15 min at 4 °C, the supernatant was filtered through a 0.2-micron membrane filter to remove any remaining particulate matter. Plasma AA levels were measured in both laboratories using the Biochrom 30PLUS AA amino acid ion exchange chromatography (IEC) system (Biochrom Ltd., Cambridge, England; https://www.biochrom.co.uk, accessed on 17 May 2024), following the procedure described by the manufacturer, which is briefly summarized below. Following the injection of 50 μL of sample into the ion-exchange chromatography system, the AAs were separated according to the standard manufacturer’s protocol using changes in ionic strength, pH of the mobile phase, and the temperature of the lithium cation exchange column with a column guard. After column separation, the samples were derivatized with ninhydrin, and the resulting colored complex was detected spectrophotometrically at 570 nm for all AAs except proline and hydroxyproline, which were detected at 440 nm. The intensity of the colored complex was proportional to the concentration of AA in the sample. Identification and quantification of AAs were based on the comparison of the retention times and the signal responses in the standard solutions with known AA concentrations, using single-point calibration. The area under the peak of each individual AA was directly proportional to its concentration. The method has a linear range of 9.8 pmoles–10 nmoles and a limit of quantitation of 15 pmol. System calibration was performed using physiological amino acids standards basics, acids, and neutrals (SIGMA Aldrich, Merck Spa, Milan, Italy). Quality control (QCs) measures were implemented using certified reference materials, specifically amino acid ERNDIM IQCS® (ERNDIM MCA Laboratory, Beatrixpark, BN, Winterswijk, The Netherlands) plasma control, lyophilized, and levels I and II for human plasma amino acids. Only analytical runs with an imprecision and inaccuracy <15% were accepted. The values obtained for all AAs from QCs fell within the certified control range.

Before combining data from patients analyzed in the two laboratories using the same assay and analytical procedure, we verified the comparability of the measurements by reviewing and comparing results from quality control (QC) materials and external quality assessment (EQAS) samples from the same batch. In addition, we assessed inter-laboratory agreement by comparing the mean concentrations of each amino acid (AA) measured in the two laboratories and found no statistically significant differences.

The plasma AAs included in this study were: alanine, arginine, asparagine, aspartate, citrulline, cystine, glutamine, glutamic acid, glycine, histidine, isoleucine, leucine, lysine, methionine, ornithine, phenylalanine, proline, serine, taurine, threonine, *α*-aminoisobutyric acid, and valine.

### 2.3 Clinical data

Demographic, clinical, instrumental, and laboratory data were retrieved retrospectively, together with anamnestic information. These included: pre/perinatal history (medication intake during pregnancy, maternal, fetal, neonatal complications, neurological signs), and family background (history of neuropsychiatric disorders). ASD severity was rated according to DSM-5 using the standardized coding system, ranging from level 1 (mild) to level 3 (severe). Core ASD symptoms were assessed with the Autism Diagnostic Observation Schedule, Second Edition (ADOS-2) (McCrimmon and Rostad, 2014), documenting both the comparison score and the module administered (modules 1, 2, or 3). In cases where ADOS-2 scores were unreliable or not classifiable due to translational issues, behavioral non-compliance, or severe motor impairments, the Childhood Autism Rating Scale, Second Edition (CARS-2) was used to provide structured symptom data (Dawkins et al., 2016).

Psychomotor delay or intellectual disability was documented, with specification of its degree, the assessment tools used (e.g., Griffiths III, WPPSI-IV), and the corresponding developmental or intellectual quotient score. Additional clinical information was extracted from caregivers’ reports and medical records, including language regression or impairment at the time of evaluation (including difficulties with pragmatics, semantics, syntax, and phonology), behavioral disturbances and hyperactivity, motor coordination difficulties and dyspraxia, comorbidity with attention deficit hyperactivity disorder (ADHD). Data on pharmacological treatments, as well as results of instrumental examinations, electroencephalogram (EEG), magnetic resonance imaging (MRI), and genetic testing, were also recorded. Dietary habits, including food selection, nutritional supplementation, and adherence to special diets, were assessed. Self-directed and outwardly directed aggressive behavior, dyspraxia, language impairment, hyperactivity, stereotypies, regression, and intellectual disability were considered as binary clinical outcomes, and the panel of 22 AAs as independent variables. All children diagnosed with ASD according to DSM-5 criteria and with the AA determination detailed above were included in the study. Exclusion criteria were: (1) diagnosis of ASD made without application of DSM-5 criteria, (2) ASD diagnosis not confirmed according to DSM-5, (3) diagnosis/presence of inherited metabolic disorders, and (4) incomplete AA results. After reviewing the preliminary dataset of 442 children, 47 subjects were excluded because the AA panel was incomplete due to missing at least one AA measurement. One additional child was excluded since they received a final diagnosis of phenylketonuria.

This study was approved by the Local Ethics Committee Lombardia 1, IRCCS San Raffaele, Milan, Italy (protocol n #CET 5–2023 of 12 July 2023, amendment approval No. CET Em. 273–2025). Informed consent was obtained from a parent or legal guardian for the treatment of anonymized data.

### 2.4 Statistical methods

Descriptive statistics were reported as the mean and standard deviation (SD) for quantitative variables and as absolute frequencies and percentages for qualitative variables. Correlations among AAs were evaluated using Spearman’s rank correlation coefficient in order to avoid multicollinearity’s problems of multivariable analyses. Indeed, AAs strongly correlated were excluded from the multivariate analysis.

To explore the underlying pattern of the AA levels, a principal component analysis (PCA) was performed and followed by a varimax rotation in order to increase the interpretability of the retained principal components. The difference in the scores of the first three rotated components between the binary clinical outcomes (i.e., self-directed and outwardly directed aggressive behavior, dyspraxia, language impairment, hyperactivity, stereotypies, regression, intellectual disability) was preliminarily assessed by a Mann–Whitney test.

In addition, a linear discriminant analysis (LDA) was performed to evaluate the discriminative ability of the first three principal components (PC1–PC3) in classifying the presence or absence of each outcome. Model performance was quantified using posterior classification probabilities, confusion matrices, and accuracy metrics, including sensitivity, specificity, and predictive values.

A parallel analysis to assess the diagnostic capacity of the level of the 12 AAs considered on the presence of each outcome has been carried out by means of a multivariable logistic regression adjusted for age and gender as baseline covariates with backward variable selection.

Model performance was assessed by estimating odds ratios (OR) with 95% confidence intervals (CIs), and the goodness of fit was assessed by the Lemeshow–Hosmer test. An OR was estimated for a unity increment (1 mmol/L) and also for a 5 and 25 mmol/L increase of the AAs. Indeed, considering the remarkable analytical variability of the laboratory measurement and the high inter-individual biological variability (Corte and Venta, 2010), for most AAs, an increase between 1 and 5 mmol/L could be considered biologically and clinically irrelevant.

Furthermore, the area under the receiver operating characteristic curve (AUC), together with its 95% CI, was estimated. Differences in AUC between nested models (baseline vs. extended with AAs) were formally tested with the paired DeLong test. An AUC threshold ≥0.65 was chosen to describe the minimally acceptable discriminatory ability of the model.

The conventional two-sided 0.05 threshold was chosen for statistical significance. Analyses were performed using SAS® 9.4 VERSION and R software (version 4.4.1, R Foundation for Statistical Computing, Vienna, Austria).

## 3 Results

### 3.1 Baseline features of the patients

Out of 442 children, 394 met the enrollment criteria. The median (25–75th percentile) age at diagnosis was 3.5 (2.8–4.6) and 3.1 (2.3–3.8) years for males and females, respectively; 81.5% were male. Their baseline features, including the frequencies of the main outcomes of the study, i.e., aggressive behavior, dyspraxia, language impairment, hyperactivity, stereotypies, regression, and intellectual disability, which included a psychomotor delay, are reported in Table 1. Of note, the low prevalence of ADHD is conditioned by the young age of the patients. Even when DSM-5 criteria for ASD were met, factors such as translational challenges (40% of children had both non-Italian parents), behavioral non-compliance, severe motor impairments, and inconsistent caregiver reports limited the estimation of ADOS-2 scores and DSM-5 severity levels. Accordingly, ADOS-2 and DSM-5 scores were available in only 54.3 and 64.5% of the case series, respectively. Additional clinical features are detailed in Supplementary Table S1. Regarding nutritional habits and special diets, two children (0.51%) had coeliac disease and were following a gluten-free diet. Food selectivity was observed in the majority of the cohort. Specifically, selectivity for meat, fruit, vegetables, and dairy products was reported in 12.0, 17.3, 18.7, and 2.0% of the children, respectively. The majority of affected children exhibited selectivity across multiple food categories, and in approximately 50% of cases, food selectivity was primarily associated with altered sensory processing and sensitivity to food texture. Owing to the low prevalence of selectivity within individual food categories, overlap, and heterogeneous nature of food selectivity, these variables could not reliably be included as covariates to adjust the analysis of AA concentrations.

**Table 1:** Established epidemiological risk factors identified in the anamnesis.

Main risk factors identified in the anamnesis	
Type	Sample size *n* (%)
Preterm birth Gestational age <37 weeks	31(7.9%)
Parental consanguinity	12 (3.0%)
Family history of ASD	72 (18.3%)
Family history of neurodevelopmental disorders	115 (29.2)
Family history of neuropsychiatric disorders	48 (12.2)

Clinical features	
Type	Sample size *n* (%)
Associated language impairment (yes)	354 (89.8%)
Dyspraxia (yes)	57 (14.5%)
Hyperactivity (yes)	144 (36.6%)
ADHD (yes)	10 (2.5%)
Hetero/self-aggressiveness (yes)	65 (16.5%)
Motor stereotypies (yes)	202 (51.3%)
Regression (yes)	123 (31.2%)
Intellectual disability (yes)	297 (75.4%)
Intellectual disability severity classification	
Mild (e.g., IQ 70–55)	58 (19.5%)
Moderate (e.g., IQ 55–40)	87 (29.3%)
Severe (e.g., IQ < 40)	38 (12.8%)
Not specified	114 (38.4%)
ASD severity according to DSM-5	
Data available	254 (64.5%)
Levels 1–2	81 (20.6%)
Level 3	173 (43.9)
ADOS-2 scores	
Data available	214 (54.3%)
<8: 32.0%	126 (32.0%)
≥8: 22.3%	88 (22.3%)

### 3.2 Principal component analysis

Accounting for the multicollinearity previously checked, plasma levels of the selected AAs, including taurine, glutamic acid, glutamine, glycine, citrulline, *α*-aminoisobutyric acid, cystine, isoleucine, tyrosine, ornithine, histidine, and arginine, were accounted for in the PCA. The first three principal components (PCs) after rotation collectively explained only 47.5% of the total variance of the data (PC1 = 21.0%, PC2 = 14.4%, and PC3 = 12.1%). The contribution (loadings) of the 12 AAs to each PC is reported in Table 2. In particular, glutamine, glycine, citrulline, tyrosine, ornithine, histidine, and arginine mainly contributed to PC1 (positive contributions); taurine, glutamic acid (positive contributions), and cystine (negative contribution) mainly contributed to PC2; and the remaining *α*-aminoisobutyric acid and isoleucine contributed to PC3. Of note, AAs involved in the urea cycle and ammonia detoxification mainly contributed to PC1, AAs linked to oxidative stress to PC2, the one branched AA to PC3, and neurotransmitters/neuromodulators to PC1 and PC2. Table 3 reports the PCs that differ significantly between children with or without a defined outcome. This result was significant only for regression, language impairment, and the presence of stereotypies. Higher levels of glutamine, glycine, citrulline, tyrosine, ornithine, histidine, arginine (grouped on PC1), taurine, glutamic acid, and low levels of cystine (grouped on PC2) were observed in children with regression vs. those without regression. Children with language impairment and with stereotypies had lower levels of glutamine, glycine, citrulline, tyrosine, ornithine, histidine, and arginine vs. those who had neither language impairment nor stereotypies. LDA did not provide evidence of the statistically significant discriminative ability of AAs grouped by PC1, PC2, and PC3 in classifying the presence or absence of each outcome. In summary, AAs involved in the urea cycle and ammonia detoxification, i.e., citrulline, ornithine, arginine, and glutamine, and some neurotransmitters/neuromodulators, i.e., glutamine, glycine, tyrosine, and histidine, mainly contributed to the first component and are significantly increased in children with regression and decreased in children with stereotypies and language impairment. AAs involved in oxidative stress/mitochondrial dysregulation, i.e., taurine, cystine (decreased), and glutamic acid, mainly contribute to the second component, which characterizes children with regression. However, none of these patterns enable discrimination between children with regression, language impairment, and stereotypies and those who do not.

**Table 2:** Loadings (%) of each AA on the first three principal components after varimax rotation.

Selected AA	PC1	PC2	PC3
Taurine	6.03	68.83*	5.24
Glutamine	58.25*	−24.28	−3.67
Glicine	60.51*	13.54	−46.98
Citrulline	59.70*	−9.48	14.37
α-aminoisobutyric acid	2.17 -	3.74	70.05*
Cystine	20.76	−54.18*	1.92
Isoleucine	33.59	20.07	74.97*
Glutamic acid	12.12	84.49*	3.91
Tyrosine	62.02*	19.98	30.46
Ornithine	**62.94**	6.60	15.90
Histidine	**38.22**	−9.28	7.98
Arginine	**59.52**	25.11	−16.91

Bold and * indicate the main contribution to the PC.

**Table 3:** Mean and SD of PC1, PC2, and PC3 for the three clinical phenotypes in which at least one of the PCs is statistically significantly different amongst the 3 PCs.

Variable	Mean (SD) in children with regression *vs.* without	Mean (SD) in children with stereotypies *vs.* without	Mean (SD) in children with language impairment *vs.* without
PC1	0.22 (1.14) *vs.* -0.1(0.92); ***p* = 0.0242**	−0.15 (0.97) *vs.* 0.26 (1.15); ***p* = 0.0020**	−0.03 (1.01) vs. 0.32 (0.88); ***p* = 0.0169**
PC2	0.08 (0.92) *vs.* -0.05 (1.04); ***p* = 0.0259**	0.07 (1.09) vs. 0.12 (0.93); *p* = 0.3183	0.03 (1) vs. -0.25 (0.96); *p* = 0.0728
PC3	0.01 (1.14) vs. -0.01 (0.94); *p* = 0.7081	0 (0.99) vs. -0.05 (1.09); *p* = 0.2625	−0.02 (1.01) vs. 0.17 (0.83); *p* = 0.4257

PC1 and PC2 patterns were significantly different (in bold) between children with regression, stereotypies, and language impairment *vs.* those without (Mann–Whitney test). For PC3, no significant difference was found.

### 3.3 Multivariable logistic regression

#### 3.3.1 Aggressive behavior (*N* = 65, 16.5%)

The increase in cystine levels was associated with aggressive behavior (*p* = 0.017); particularly, a 1 mmol/L increase gives an OR = 1.03 (95%CI: 1.01; 1.06) and a 25 mmol/L increase gives an OR = 2.13 (95%CI: 1.15; 3.97). However, the inclusion of cystine in the basal model with gender and age did not significantly increase the AUC, which was 0.64 (95%CI: 0.56; 0.71).

#### 3.3.2 Language impairment (*N* = 354, 89.8%)

**Figure 1:**
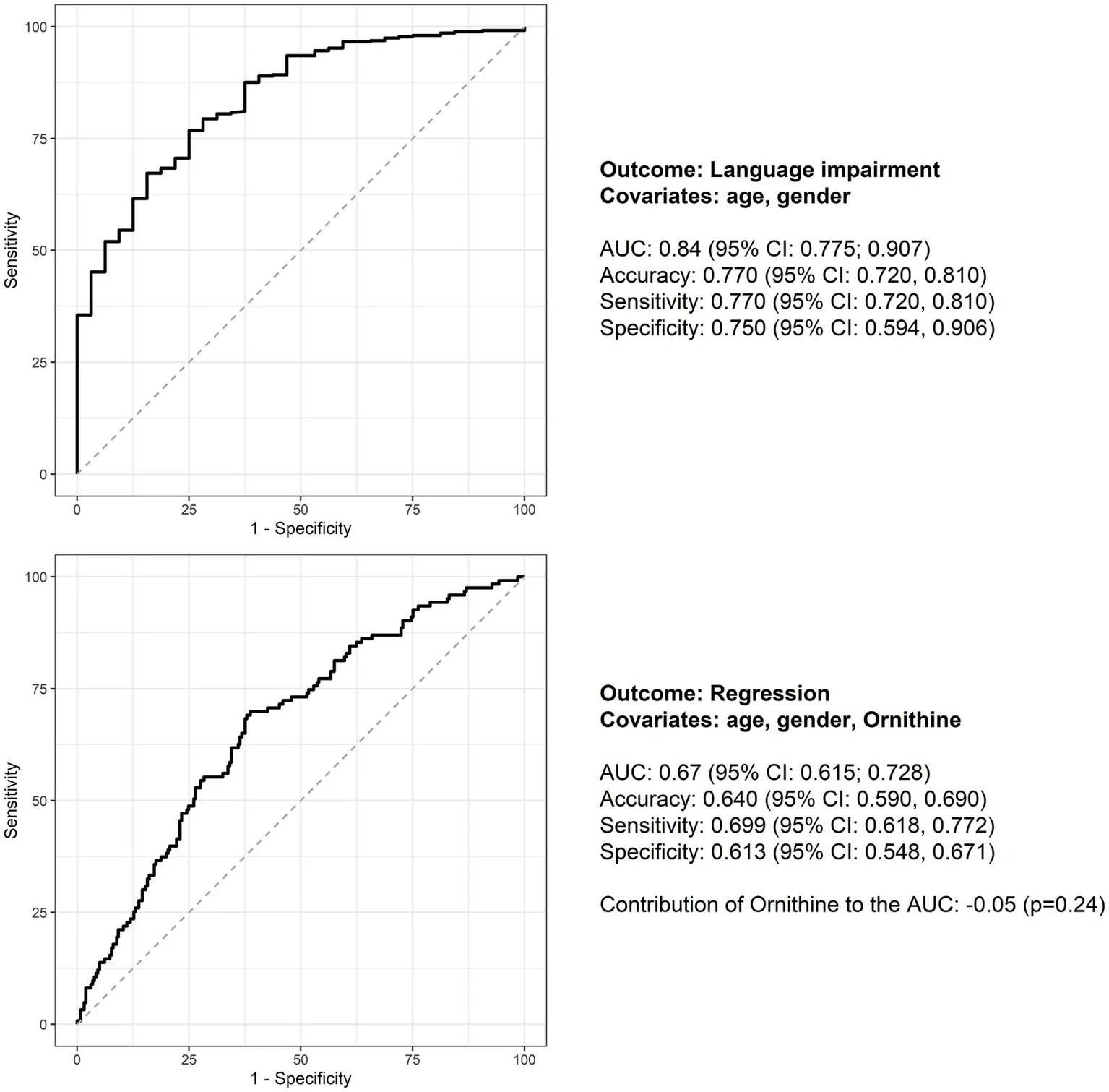
Discriminative performance of the multivariable logistic regression models, expressed as the area under the receiver operating characteristic curve (AUC-ROC), for the outcomes language impairment **(A)** and regression **(B)**.

No significant associations with AAs were found. The baseline model including only gender and age gave an AUC of 0.84 (95%CI: 0.78; 0.91) (Figure 1A).

#### 3.3.3 Dyspraxia (*N* = 57, 14.5%)

No significant associations with AAs were found.

#### 3.3.4 Hyperactivity (*N* = 144, 36.6%)

Hyperactivity was inversely associated with the increase in arginine levels (*p* = 0.0023), with an OR = 0.99 (95%CI: 0.98; 0.99) for a 1 mmol/L increase and an OR = 0.70 (95%CI: 0.55; 0.88) for a 25 mmol/L increase. Cystine level increase was directly associated with hyperactivity (*p* = 0.0182), with an OR = 1.02 (95%CI: 1; 1.04) for a 1 mmol/L increase and an OR = 1.79 (95%CI: 1.1; 2.9) for a 25 mmol/L increase. Taurine was also directly associated with hyperactivity (*p* = 0.0121), with an OR = 1.01 (95% CI: 1; 1.02) for a 1 mmol/L increase and an OR = 1.24 (95% CI: 1.05; 1.46) for a 25 mmol/L increase. The AUC for the complete model including the three AAs was 0.63 (95%CI: 0.57; 0.69), but only cystine and marginally taurine significantly increased the AUC of the baseline model (*p* = 0.0286 and *p* = 0.0569, respectively). After the removal of arginine, the final AUC was 0.59 (95%CI: 0.53; 0.65), and the contribution of cystine and taurine remained unchanged.

#### 3.3.5 Regression (*N* = 123, 31.2%)

Age was inversely associated with regression (*p* < 0.0001), with an OR = 0.73 (95%CI: 0.63; 0.85) for a 1-year increase. Ornithine was directly associated with regression (*p* = 0.0046), with an OR = 1.02 (95%CI: 1.01; 1.03) for a 1 mmol/L increase and an OR = 1.5 (95%CI: 1.13; 1.98) for a 25 mmol/L increase. The AUC for the complete model was 0.67 (95%CI: 0.62; 0.73), and the addition of ornithine did not significantly contribute to the baseline model (Figure 1B).

#### 3.3.6 Stereotypies (*N* = 202, 51.3%)

**Figure 2:**
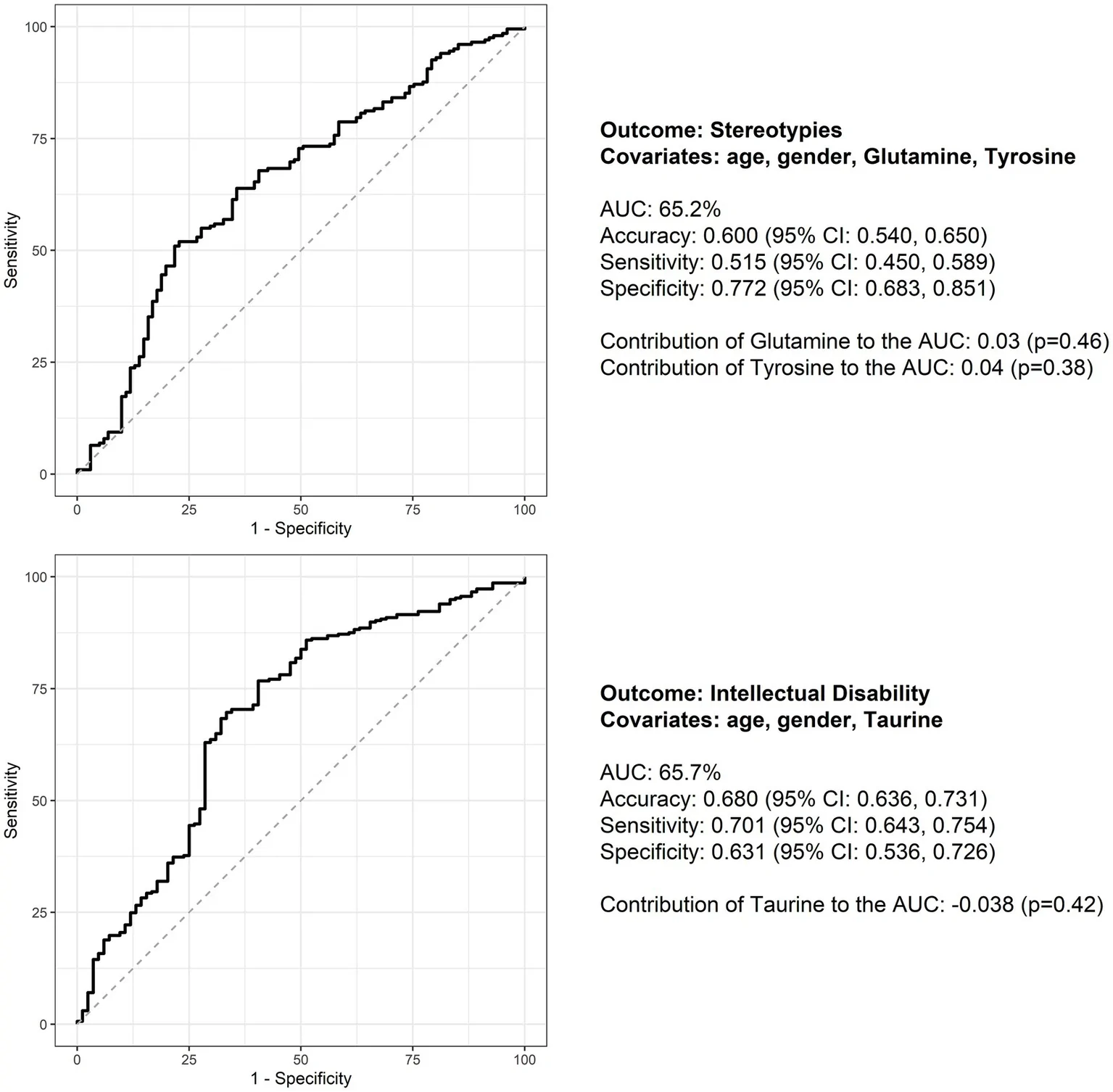
Discriminative performance of the multivariable logistic regression models, expressed as the area under the receiver operating characteristic curve (AUC-ROC), for the outcomes of stereotyped behavior **(A)** and intellectual disability **(B)**.

The increase of cystine was directly associated with the presence of stereotypies (*p* = 0.0143), with an OR = 1.03 (95%CI: 1.01; 1.05) for a 1 mmol/L increase and an OR = 2.07(95%CI: 1.16; 3.70) for a 25 mmol/L increase. Conversely, the increase in glutamine was inversely associated with the presence of stereotypies (*p* = 0.0122), with an OR = 1 (95%CI: 0.99, 1) for a 1 mmol/L increase and an OR = 0.90 (95%CI: 0.84; 0.98) for a 25 mmol/L increase. Similarly, tyrosine was inversely associated with the presence of stereotypies (*p* = 0.0153), with an OR = 0.99 (95%CI: 0.97; 1) for a 1 mmol/L increase and an OR = 0.70 (95%CI: 0.52; 0.93) for a 25 mmol/L increase. The AUC of the full model including the 3 AAs was 0.65 (95%CI: 0.59; 0.72) but only glutamine and tyrosine significantly contributed to the discriminatory capability of the baseline model (*p* = 0.0042 and *p* = 0.0021, respectively). However, the removal of cystine turned the contribution of glutamine and tyrosine into not statistically significant (Figure 2A).

#### 3.3.7 Intellectual disability (*N* = 297, 75.4%)

The increase in age was inversely associated with intellectual disability, with an OR = 0.84 (95%CI: 0.75; 0.93) for 1 year of age.

The increase in taurine was directly associated with intellectual disability (*p* = 0.0271), with an OR = 1.01 (95%CI: 1.00; 1.02) for a 1 mmol/L increase and an OR = 1.29 (95%CI: 1.03; 1.63) for a 25 mmol/L increase. The full model was associated with an AUC of 0.66 (95%CI:0.58; 0.73), and the contribution of taurine to the baseline model including age and gender did not significantly increase the AUC (Figure 2B).

A schematic summary of the relationship between AA variations and outcomes is reported in Table 4.

**Table 4:** Association between AA increase/decrease, outcome, and possible pathophysiological insights.

AA	OR	Outcome	Possible pathophysiological insights
**↑**Cystine	**↑**	Stereotypies Hyperactivity Aggressive behavior	Oxidative stress
**↑**Taurine	**↑**	Hyperactivity Intellectual disability	Oxidative stress and intestinal dysbiosis
**↑**Ornithine	(marginally)↑	Regression	Ammonia detoxification (possible block in the arginine conversion pathway)
**↑**Arginine	**↓**	Hyperactivity	Ammonia detoxification
**↑**Glutamine **↑**Tyrosine	**↓** **↓**	Stereotypies	Neuroprotection

## 4 Discussion

Current literature suggests that children with ASD exhibit altered AA profiles; however, findings regarding increases or decreases in specific AA concentrations remain inconsistent, likely due to age-related metabolic changes, heterogeneity across ASD cohorts, and the limitations of the predominantly univariate statistical approaches used in most studies (Ferraro et al., 2025; Li et al., 2025; James et al., 2004; Chang et al., 2024). The ultimate aim of investigating AA abnormalities as potential metabolic signatures of ASD is to identify therapeutic targets (Smith et al., 2019; Ahmed, 2025) that may contribute to symptom mitigation. Achieving this goal, however, requires a thorough assessment of the relationships between AA patterns and clinical phenotypes.

To date, only a limited number of studies (Li et al., 2025) including the present investigation have addressed this question using robust statistical frameworks and multivariate analytical methods, considering the pragmatic use of AA testing in the clinical context.

By analyzing comprehensive clinical data from a large cohort of very young children at the time of ASD diagnosis, our study identified a protective association of increased tyrosine and glutamine levels with the presence of stereotyped behaviors, consistent with their established role in modulating neurotransmission. Tyrosine is involved in dopamine-related pathways, and tyrosine supplementation may mitigate the negative side-effects of dopamine-related pathologies such as Parkinson’s disease (Jongkees et al., 2014; Growdon et al., 1982). The increase in glutamine likely reflects a more balanced glutamate (excitatory) and GABA (inhibitory) neurotransmission, which is often reported to be disrupted in subjects with ASD and motor stereotypies (Harris et al., 2016; Rojas, 2014; Zou et al., 2010). Nonetheless, the protective effect of glutamine increase may be related to the crucial role of cytosolic glutamine synthetase for ammonia removal in the brain, under conditions of ammonia stress (Zhou et al., 2020). However, ammonia derived from glutamine can disrupt mitochondrial function by increasing reactive oxygen species and causing mitochondrial permeability, leading to impaired ATP synthesis and oxidative stress (Lemberg and Fernández, 2009). This may provide an explanation for our finding of a positive association between the presence of stereotyped behaviors and increased cystine levels, as cystine is a recognized hallmark of oxidative stress. Cystine is a precursor of glutathione and taurine, both of which exert neuroprotective and antioxidant functions, and N-acetylcysteine supplementation has shown promise in reducing irritability and hyperactivity in ASD, likely by enhancing Glutathione availability (Lee et al., 2021). In addition to cystine, we have also demonstrated that taurine elevation is positively associated with hyperactivity, whereas Arginine increase represents a protective factor. These findings reinforce the hypothesis that in these children cystine and taurine rising may reflect a compensatory response to oxidative stress, also enhanced by ammonia detoxification (arginine increase), which is an additional pathophysiological mechanism. Furthermore, intellectual disability appeared to be triggered by metabolic alterations linked to the increase in oxidative stress, according to the increase in taurine. Notably, in children with ASD, high levels of free taurine and unconjugated bile acids may result from deconjugation of bile acids by enteric bacteria in the gastrointestinal tract (Needham et al., 2021; Duszka, 2022). This bacterial activity alters primary bile acids, releasing the AAs glycine or taurine, which are then free in the intestine (Guzior and Quinn, 2021; Wong et al., 2021; Hofmann, 1999). Finally, increased ornithine levels are positively (but only marginally) associated with the presence of regression. Previous studies have linked ornithine to altered psychometric outcomes (poorer receptive language, reduced socialization, stereotypic behaviors, and motor deficits) (Wang et al., 2022). Since ornithine is a precursor for arginine synthesis and is produced via arginine breakdown, its elevation might indicate impaired ammonia detoxification. Of note, the overall discriminatory ability of AA concentrations is too low to be considered in the diagnostic pathway of ASD.

Our study has several strengths, including a large sample size, a young age of participants, a comprehensive collection of clinical covariates, and a multivariate statistical approach. Nevertheless, some limitations should be acknowledged.

First, we were unable to include an age-matched control group, as AA determinations are routinely recommended and performed only in children presenting with specific neurological, neuropsychiatric, or metabolic conditions.

Second, plasma AA concentrations may be influenced by multiple factors, including dietary intake, food selectivity, nutritional supplementation, medication use, and overall nutritional status, all of which are particularly difficult to characterize comprehensively in children with ASD. Consequently, we were unable to perform sensitivity analyses to account for these potential confounders. In addition, data on parental education and household socioeconomic status were unavailable, precluding investigation of associations with potentially relevant sociodemographic variables.

Another limitation is the lack of data on tryptophan, an AA of particular interest in ASD research, because the analytical platform used was not designed to quantify individual kynurenine pathway metabolites. Furthermore, to maximize the sample size, we combined the AA measurements obtained from two laboratories after confirming their analytical comparability. However, this approach required restricting the AA panel to those routinely measured by both laboratories, as one laboratory analyzed a smaller set of AAs.

Finally, although the young age of the participants represents a major strength of this cohort, it may also have affected the estimation of ADOS-2 scores and DSM-5 severity levels. Additional factors, including translation-related issues, behavioral non-compliance, severe motor impairments, and inconsistencies in caregiver reports, may have further limited the reliability of these assessments (Diagnostic and Statistical Manual of Mental Disorders, 2026). While DSM-5 provides a robust diagnostic framework, its severity specifiers are based on support needs and therefore should not be considered quantitative measures of symptom severity. Similarly, although children may meet DSM-5 criteria for ASD, ADOS-2 scores may be difficult to interpret or may not be valid when standardized administration cannot be ensured. This may occur in the presence of translation challenges, extremely limited developmental abilities, refusal to engage, marked distress or behavioral dysregulation, severe sensory or motor impairments, or atypical developmental profiles, particularly among very young children (Lord et al., 2012; Zander et al., 2016; Gotham et al., 2009; Adamou et al., 2021).

In conclusion, our findings seem to suggest that oxidative stress (taurine and cystine alterations), intestinal dysbiosis (taurine increase), urea-cycle dysregulation (altered arginine, ornithine, and glutamine), excitotoxicity (glutamine imbalance), and altered neurotransmission (altered glutamine and tyrosine) may have a role in young children with ASD and aggressive behavior, regression, stereotypies, hyperactivity, and intellectual disability. These findings have to be regarded as exploratory and hypothesis-generating. While they may inform future research, they should not be interpreted as evidence of clinically meaningful predictive performance, specific metabolic signatures of ASD, or causal biological mechanisms.

Nonetheless, the clinical interpretation and application of AA measurements remain highly challenging. The marked heterogeneity of the ASD population, together with substantial inter-individual variability in AA concentrations, currently precludes the definition of reliable cutoffs or reference ranges that could be used to identify specific clinical phenotypes or guide therapeutic interventions (Corte and Venta, 2010). At present, AA profiling has limited diagnostic utility in children with ASD and remains associated with considerable costs.

This study should be considered a preliminary effort to develop a cost-effective AA profiling strategy based on a selected set of biomarkers with independent clinical value. Together with an improved understanding of the biological and clinical implications of AA alterations, such an approach may enhance the identification of metabolically distinct subgroups and contribute to the development of earlier and more personalized interventions. In this context, personalized clinical and nutritional strategies may represent a promising avenue for the early management of ASD, a complex neurodevelopmental condition for which pharmacological treatment options remain limited (Kostic and Buxbaum, 2021).

## Data Availability

The raw data supporting the conclusions of this article will be made available by the authors, without undue reservation.

## References

[ref1] Adamou M. Jones S. L. Wetherhill S. (2021). Predicting diagnostic outcome in adult autism spectrum disorder using the autism diagnostic observation schedule, second edition. BMC Psychiatry 21:24. doi: 10.1186/s12888-020-03028-7, 33423664 PMC7798323

[ref2] Ahmed H. S. (2025). The multifaceted role of L-type amino acid transporter 1 at the blood-brain barrier: structural implications and therapeutic potential. Mol. Neurobiol. 62, 3813–3832. doi: 10.1007/s12035-024-04506-9, 39325101

[ref3] Arum P. Amareta D. I. Zannah F. (2017). Phenylalanine and tryptophan intake of hyperactive children with autism. J. Biomed. Transl. Res. 3, 34–36. doi: 10.14710/jbtr.v3i2.1744

[ref4] Ashe K. Kelso W. Farrand S. Panetta J. Fazio T. De Jong G. . (2019). Psychiatric and cognitive aspects of phenylketonuria: the limitations of diet and promise of new treatments. Front. Psych. 10:561. doi: 10.3389/fpsyt.2019.00561, 31551819 PMC6748028

[ref5] Campistol J. Díez-Juan M. Callejón L. Fernandez-De Miguel A. Casado M. Garcia Cazorla A. . (2016). Inborn error metabolic screening in individuals with nonsyndromic autism spectrum disorders. Dev. Med. Child Neurol. 58, 842–847. doi: 10.1111/dmcn.13114, 27038397

[ref6] Chang X. Zhang Y. Chen X. Li S. Mei H. Xiao H. . (2024). Gut microbiome and serum amino acid metabolome alterations in autism spectrum disorder. Sci. Rep. 14:4037. doi: 10.1038/s41598-024-54717-2, 38369656 PMC10874930

[ref7] Corte Z. Venta R. (2010). Biological variation of free plasma amino acids in healthy individuals. Clin. Chem. Lab. Med. 48, 99–104. doi: 10.1515/CCLM.2010.008, 19929754

[ref8] Cross-Disorder Group of the Psychiatric Genomics Consortium (2019). Genomic relationships, novel loci, and pleiotropic mechanisms across eight psychiatric disorders. Cell 179, 1469–1482.e11. doi: 10.1016/j.cell.2019.11.020 31835028 PMC7077032

[ref9] Dawkins T. Meyer A. T. Van Bourgondien M. E. (2016). The relationship between the childhood autism rating scale: second edition and clinical diagnosis utilizing the DSM-IV-TR and the DSM-5. J. Autism Dev. Disord. 46, 3361–3368. doi: 10.1007/s10803-016-2860-z, 27422400

[ref10] Diagnostic and Statistical Manual of Mental Disorders. (2026). Psychiatry Online. Available online at: https://psychiatryonline.org/doi/book/10.1176/appi.books.9780890425596 (Accessed June 18, 2026).

[ref11] Duszka K. (2022). Versatile triad alliance: bile acid, taurine and microbiota. Cells 11:2337. doi: 10.3390/cells11152337, 35954180 PMC9367564

[ref12] Ferraro S. Saielli L. Biganzoli D. Tosi M. Guidi L. Longo R. . (2025). Amino acid patterns in children with autistic Spectrum disorder: a preliminary biochemical evaluation. Nutrients 17:274. doi: 10.3390/nu17020274, 39861405 PMC11767892

[ref13] Gotham K. Pickles A. Lord C. (2009). Standardizing ADOS scores for a measure of severity in autism spectrum disorders. J. Autism Dev. Disord. 39, 693–705. doi: 10.1007/s10803-008-0674-3, 19082876 PMC2922918

[ref14] Growdon J. H. Melamed E. Logue M. Hefti F. Wurtman R. J. (1982). Effects of oral L-tyrosine administration on CSF tyrosine and homovanillic acid levels in patients with Parkinson’s disease. Life Sci. 30, 827–832. doi: 10.1016/0024-3205(82)90596-3, 6175872

[ref15] Guzior D. V. Quinn R. A. (2021). Review: microbial transformations of human bile acids. Microbiome 9:140. doi: 10.1186/s40168-021-01101-1, 34127070 PMC8204491

[ref16] Harris A. D. Singer H. S. Horska A. Kline T. Ryan M. Edden R. a. E. . (2016). GABA and glutamate in children with primary complex motor stereotypies: an 1H-MRS study at 7T. AJNR Am. J. Neuroradiol. 37, 552–557. doi: 10.3174/ajnr.A4547, 26542237 PMC4792666

[ref17] Hirota T. King B. H. (2023). Autism Spectrum disorder: a review. JAMA 329, 157–168. doi: 10.1001/jama.2022.23661, 36625807

[ref18] Hofmann A. F. (1999). The continuing importance of bile acids in liver and intestinal disease. Arch. Intern. Med. 159, 2647–2658. doi: 10.1001/archinte.159.22.2647, 10597755

[ref19] Hyman S. L. Levy S. E. Myers S. M. (2020). Council on Children with Disabilities Section on Developmental and behavioral pediatrics identification, evaluation, and management of children with autism spectrum disorder. Pediatrics 145:e20193447. doi: 10.1542/peds.2019-3447, 31843864

[ref20] James S. J. Cutler P. Melnyk S. Jernigan S. Janak L. Gaylor D. W. . (2004). Metabolic biomarkers of increased oxidative stress and impaired methylation capacity in children with autism. Am. J. Clin. Nutr. 80, 1611–1617. doi: 10.1093/ajcn/80.6.1611, 15585776

[ref21] Jongkees B. J. Hommel B. Colzato L. S. (2014). People are different: tyrosine’s modulating effect on cognitive control in healthy humans may depend on individual differences related to dopamine function. Front. Psychol. 5:1101. doi: 10.3389/fpsyg.2014.01101, 25339925 PMC4186281

[ref22] Kostic A. Buxbaum J. D. (2021). The promise of precision medicine in autism. Neuron 109, 2212–2215. doi: 10.1016/j.neuron.2021.06.025, 34293291

[ref23] Lee T.-M. Lee K.-M. Lee C.-Y. Lee H.-C. Tam K.-W. Loh E.-W. (2021). Effectiveness of N-acetylcysteine in autism spectrum disorders: a meta-analysis of randomized controlled trials. Aust. N. Z. J. Psychiatry 55, 196–206. doi: 10.1177/0004867420952540, 32900213

[ref24] Lemberg A. Fernández M. A. (2009). Hepatic encephalopathy, ammonia, glutamate, glutamine and oxidative stress. Ann. Hepatol. 8, 95–102. doi: 10.1016/S1665-2681(19)31785-5, 19502650

[ref25] Li J. Zhai P. Bi L. Wang Y. Yang X. Yang Y. . (2025). Associations between amino acid levels and autism spectrum disorder severity. BMC Psychiatry 25:332. doi: 10.1186/s12888-025-06771-x, 40186136 PMC11969702

[ref26] Lombardy Regional Executive Committee. (2026). I disturbi dello spettro autistico. Available online at: https://www.regione.lombardia.it/sanita/cure-specialistiche/i-disturbi-dello-spettro-autistico (Accessed June 18, 2026)

[ref27] Lord C. Rutter M. DiLavore P. C. Risi S. Gotham K. Bishop S. (2012). Autism Diagnostic Observation Schedule. 2nd Edn Torrance: Western Psychological Services, Scientific Research Publishing. doi: 10.1177/0734282913490916

[ref28] Maenner M. J. Warren Z. Williams A. R. Amoakohene E. Bakian A. V. Bilder D. A. . (2023). Prevalence and characteristics of autism Spectrum disorder among children aged 8 years - autism and developmental Disabilities monitoring network, 11 sites, United States, 2020. MMWR Surveill. Summ. 72, 1–14. doi: 10.15585/mmwr.ss7202a1, 36952288 PMC10042614

[ref29] Maynard T. M. Manzini M. C. (2017). Balancing act: maintaining amino acid levels in the autistic brain. Neuron 93, 476–479. doi: 10.1016/j.neuron.2017.01.015, 28182903

[ref30] McCrimmon A. Rostad K. (2014). Test review: autism diagnostic observation schedule, second edition (ADOS-2) manual (part II): toddler module. *Journal of Psychoeducational Assessment* 32, 88–92.

[ref31] Needham B. D. Adame M. D. Serena G. Rose D. R. Preston G. M. Conrad M. C. . (2021). Plasma and fecal metabolite profiles in autism Spectrum disorder. Biol. Psychiatry 89, 451–462. doi: 10.1016/j.biopsych.2020.09.025, 33342544 PMC7867605

[ref32] Rojas D. C. (2014). The role of glutamate and its receptors in autism and the use of glutamate receptor antagonists in treatment. J. Neural Transm. 121, 891–905. doi: 10.1007/s00702-014-1216-0, 24752754 PMC4134390

[ref33] Sanders S. J. He X. Willsey A. J. Ercan-Sencicek A. G. Samocha K. E. Cicek A. E. . (2015). Insights into autism Spectrum disorder genomic architecture and biology from 71 risk loci. Neuron 87, 1215–1233. doi: 10.1016/j.neuron.2015.09.016, 26402605 PMC4624267

[ref34] Schiff M. Benoist J.-F. Aïssaoui S. Boespflug-Tanguy O. Mouren M.-C. de Baulny H. O. . (2011). Should metabolic diseases be systematically screened in nonsyndromic autism spectrum disorders? PLoS One 6:e21932. doi: 10.1371/journal.pone.0021932 21760924 PMC3131397

[ref35] Smith A. M. King J. J. West P. R. Ludwig M. A. Donley E. L. R. Burrier R. E. . (2019). Amino acid dysregulation Metabotypes: potential biomarkers for diagnosis and individualized treatment for subtypes of autism Spectrum disorder. Biol. Psychiatry 85, 345–354. doi: 10.1016/j.biopsych.2018.08.016, 30446206 PMC6837735

[ref36] Smon A. Cuk V. Brecelj J. Murko S. Groselj U. Zerjav Tansek M. . (2019). Comparison of liquid chromatography with tandem mass spectrometry and ion-exchange chromatography by post-column ninhydrin derivatization for amino acid monitoring. Clin. Chim. Acta 495, 446–450. doi: 10.1016/j.cca.2019.05.007, 31077651

[ref37] Wang L. Zheng R. Xu Y. Zhou Z. Guan P. Wu Y. . (2022). Altered metabolic characteristics in plasma of young boys with autism Spectrum disorder. J. Autism Dev. Disord. 52, 4897–4907. doi: 10.1007/s10803-021-05364-3, 34800227

[ref38] Wong G. C. Montgomery J. M. Taylor M. W. (2021). “The gut-microbiota-brain Axis in autism Spectrum disorder,” in Autism Spectrum Disorders, ed. Grabrucker A. M. (Brisbane, AU: Exon Publications). 34495615

[ref39] Yektaş Ç. Alpay M. Tufan A. E. (2019). Comparison of serum B12, folate and homocysteine concentrations in children with autism spectrum disorder or attention deficit hyperactivity disorder and healthy controls. Neuropsychiatr. Dis. Treat. 15, 2213–2219. doi: 10.2147/NDT.S212361, 31496704 PMC6689552

[ref40] Zander E. Willfors C. Berggren S. Choque-Olsson N. Coco C. Elmund A. . (2016). The objectivity of the autism diagnostic observation schedule (ADOS) in naturalistic clinical settings. Eur. Child Adolesc. Psychiatry 25, 769–780. doi: 10.1007/s00787-015-0793-2, 26584575

[ref41] Zheng Z. Zhu T. Qu Y. Mu D. (2016). Blood glutamate levels in autism Spectrum disorder: a systematic review and Meta-analysis. PLoS One 11:e0158688. doi: 10.1371/journal.pone.0158688, 27390857 PMC4938426

[ref42] Zhou Y. Eid T. Hassel B. Danbolt N. C. (2020). Novel aspects of glutamine synthetase in ammonia homeostasis. Neurochem. Int. 140:104809. doi: 10.1016/j.neuint.2020.104809, 32758585

[ref43] Žigman T. Petković Ramadža D. Šimić G. Barić I. (2021). Inborn errors of metabolism associated with autism Spectrum disorders: approaches to intervention. Front. Neurosci. 15:673600. doi: 10.3389/fnins.2021.673600, 34121999 PMC8193223

[ref44] Zou J. Wang Y.-X. Dou F.-F. Lü H.-Z. Ma Z.-W. Lu P.-H. . (2010). Glutamine synthetase down-regulation reduces astrocyte protection against glutamate excitotoxicity to neurons. Neurochem. Int. 56, 577–584. doi: 10.1016/j.neuint.2009.12.021, 20064572 PMC2831119

