# Supplementary material for: Amino acid profiles and clinical phenotypes in young children with autism spectrum disorder
Source: Front Neurosci. 2026 Sep 3;20:1913701. doi: 10.3389/fnins.2026.1913701 (PMC13582212; doi:10.3389/fnins.2026.1913701)
Supplement: Supplementary file 1 [file Data_Sheet_1.docx]

**Figure 1S.** **Correlation matrix showing the correlation coefficients between AAs.**

**Highlighted in yellow those statistically significant.**

**Table 1 S Additional clinical features of the investigated children**

| **Pre/Perinatal History** | |
| --- | --- |
| **Data Available** | 335 (85%) |
| **Complicated^a^** | 128 (32.5%) |
| **Main reported factors** | |
| **Maternal Gestational Diabetes** | 27 (6.9%) |
| **Medication Use During Pregnancy** | |
| **Data Available** | 391 (99.2%) |
| **Yes** | 32 (8.1%) |
| **Main reported Medications** | |
| **Insulin** | 7 (1.8%) |
| Medications for thyroid disorders | 11 (2.8) |
| **Neonatal Clinical Conditions** | |
| **Data Available** | 389 (98.7%) |
| **Complicated^b^** | 54 (13.7%) |
| **Neurological Examination at Birth** 388 (98.5%) | |
| Neurological signs | 36 (8.9%) |
| **Type** | |
| Hypotonia | 24(66.7%) |
| Pyramidal Signs | 6 (1.6%) |
| Cerebellar Signs | 1 (0,3%) |
| Extrinsic Ocular Motility | 2 (0,5%) |
| Others | 3 (0,8%) |
| **Clinical Features** | |
| **Epilepsy** | |
| **Data Available** | **388 (98.5%)** |
| Yes | 16 (4.1%) |
| **Facial Dysmorphisms** | |
| **Data Available** | **382 (97%)** |
| Yes | 36 (9.4%) |
| **Other Malformations** | |
| Cardiac Malformation | 2 (0.5%) |
| Gastrointestinal (GI) Malformation | 2 (0.5%) |
| Genitourinary (GU) Malformation | 7 (1.8%) |
| Skeletal Malformation | 4 (0.9%) |
| **Microcephaly** | |
| No | 379 (96.2%) |
| Yes | 15 (3.8%) |
| **Macrocephaly, n (%)** | |
| **Data Available** | 394 (100%) |
| Yes | 26 (6.6%) |
| **Food Selectivity** | |
| **Data Available** | 382 (96.9%) |
| Yes | 174 (45.5%) |
| **Food Selectivity Type (tentatively defined in 150 subjects on 175)** | |
| Meat | 18 (12%) |
| Fruit | 26 (17.3%) |
| Vegetables | 28 (18.7%) |
| Dairy Products | 3 (2%) |
| Due to altered sensory processing/food texture* | 75 (50%) |
| **Sleep-Wake Cycle Abnormalities** | |
| **Data Available** | 381 (96.7%) |
| Yes | 110 (28,9%) |
| **Sleep-Wake Cycle Abnormalities description (available in 105 on 110 subjects)** | |
| **Circadian Rhythm Disturbances** | 6 (1.6%) |
| **Initial Insomnia (Difficulty Falling Asleep)** | 47 (11.9%) |
| **Middle Insomnia (Frequent Night Wakings)** | 49 (12.4%) |
| **Terminal Insomnia** | 3 (0.8%) |
| **IMAGING** | |
| **EEG,** **n (%)** | |
| **Data Available** | 353 (89.6%) |
| N**ormal** | 293 (74.4%) |
| **Epileptiform Abnormalities** | 60 (15.2%) |
| **Brain MRI, n (%)** | |
| **Data Available** | 298 (75.6%) |
| **Normal** | 267 (67.8%) |
| **Abnormal** | 31 (7.9%) |
| *Details:* |  |
| **Cortical Abnormalities** | 2 (0.5%) |
| **Corpus Callosum Dysmorphisms** | 12 (3.0%) |
| **Cerebellar Abnormalities** | 2 (0.5%) |
| **Others** | 15 (3.5%) |

***Percentages are estimated on number of children with selectivity. More patterns may be present in each child.**

**Table 2S Median(25-75^th^) concentrations (mmol/L) of the selected AA in the 394 children.**

| **Variables** | **N = 394** |
| --- | --- |
| **Taurine** |  |
| *Median (Q1, Q3)* | 70 (56.0, 90) |
| **Glutamic Acid** |  |
| *Median (Q1, Q3)* | 46 (34.0, 62) |
| **Glutamine** |  |
| *Median (Q1, Q3)* | 476 (423.0, 522) |
| **Glicine** |  |
| *Median (Q1, Q3)* | 211 (183.0, 242) |
| **Citrulline** |  |
| *Median (Q1, Q3)* | 30 (25.0, 37) |
| **Alpha-aminoisobutyric Acid** |  |
| *Median (Q1, Q3)* | 18 (14.0, 23) |
| **Cystine** |  |
| *Median (Q1, Q3)* | 24 (17.0, 33) |
| **Isoleucine** |  |
| *Median (Q1, Q3)* | 59 (51.0, 70) |
| **Tyrosine** |  |
| *Median (Q1, Q3)* | 68 (58.0, 81) |
| *Unknown* | 53 |
| **Ornithine** |  |
| *Median (Q1, Q3)* | 57 (46.0, 74) |
| **Histidine** |  |
| *Median (Q1, Q3)* | 77 (68.0, 84) |
| **Arginine** |  |
| *Median (Q1, Q3)* | 70 (55.0, 87) |
